# Remarks on Vaccination; on Its Value as a Preventative of Small Pox, and the Causes of Its Occasional Failure

**Published:** 1830

**Authors:** James C. Finley


ART.—II. *Remarks on vaccination; on its value as a preventative of small pox, and on the causes of its occasional failure.* By JAMES C. FINLEY, M. D.

The great extent to which small pox has prevailed in this city, during the past year, the fatality it has occasioned, and the numerous cases reported to have occurred after vaccination, have excited a great deal of solicitude upon this subject. The numerous and anxious enquiries made respecting the protective influence of the kind pox, and the causes of its failure, will justify us in inviting the attention of the profession to facts which are so fully within their reach, that ignorance is inexcusable.

We have nothing to offer, either original or novel. The subject has been so fully investigated as to leave scarcely any thing for the ingenuity of speculation ; yet in this as in many other cases, the very simplicity of the subject, and the facility of acquiring information, have led to a very culpable negligence.

We shall first lay down the characteristics of the genuine vaccine disease, and then proceed to enquire into the causes local or constitutional which lead to its failure ; the extent of its protective influence ; its efficacy in this respect compared with small pox ; and lastly into the value of the various precautions which have been proposed for the more effectual security of the system against the attacks of small pox.

1. The genuine vesicle appears on the third or fourth day after vaccination—it is less satisfactory if the inflammation commence earlier. The vesicle when complete is circular, or at least oval, with regular and well defined edges ; cellulose ; of a pearl colour with a depression in the centre of a darker colour, surrounded by an areola with some degree of swelling and hardness ; and containing a fluid clear and transparent, which concretes after the 12th day into a hard scab, semi-transparent and of a mahogany colour. To afford effectual security, the progress of the disease should be uninterrupted by extraneous or constitutional irritation, and the scab should be allowed to remain undisturbed until the 21st day.

The scar should be circular, or at least oval with regular and well defined out lines ; permanently depressed below the level of the surrounding skin ; not larger than a small wafer ; and it is more satisfactory if it be indented and radiated.

To be fully satisfactory, the local disease should be accompanied with slight constitutional symptoms—general fever, and enlargement of the lymphatic glands ; and the glands are generally more enlarged than from a corresponding degree of local inflammation arising from any other cause. This circumstance is to be particularly attended to ; for there is reason to believe, that the disease is sometimes strictly local, affording no protection to the system, and that many of the cases of subsequent small pox are to be attributed to this fact.

The sources of imperfection are ; 1. Effete or vitiated virus. It has been supposed that the virus gradually loses its specific character, by passing through successive individuals who labored under some constitutional or accidental inaptitude to the disease ; thus rendering necessary an occasional recurrence to the original source for a fresh supply. Later and more extended observation however, has shown that where the imperfection of the vesicle arises from constitutional inaptitude, the virus may still in a healthy individual produce the vaccine disease in its most perfect form, bearing in this respect an analogy to the modified small pox ; the virus of which, although the distinctive characters of the disease are scarcely any longer to be recognized, will reproduce small pox in its most virulent forms. As the vaccine lymph therefore, after having passed through hundreds of individuals, still produces the original disease with all its features, it cannot be admitted that it is liable, in this way, to lose any thing of its original efficacy. Nevertheless, it will not be denied that by successive inocculations in persons laboring under this incompatibility, it may at length lose its specific character ; or that when taken from a vesicle modified by some contagious cutaneous disease, it may carry with it a source of irritation that will produce a similar modification in the person who receives it. The lymph also, we may add, loses its contagious properties by age and exposure ;—the difficulty of communicating the infection is hereby increased, though the protection afforded, when the disease is excited, remains the same.

2. Other sources of imperfection are ; various acute diseases, as fever, visceral inflammation, &c. ; hooping cough ; dentition ; the irritation of the sore by rubbing, or by puncturing for the purpose of procuring lymph; and lastly every variety of cutaneous diseases. So generally is the disease modified by the last of these causes, that we have never witnessed a case of successful vaccination where a child, apparently healthy in other respects, was affected with the slightest eruptive disease.

3. To these causes we may perhaps add a scrofulous diathesis when strongly marked, general ill health, and an advanced period of life.

4. There is occasionally an inaptitude for the vaccine disease, with persons in perfect health, which continues through a series of years, and probably in some instances through life. We have met with two instances within the last six months in which, repeated vaccination both from the scab and the vesicle produced no effect, except in one instance to produce after numerous trials, a papulous efflorescence on the second day, which disappeared on the fifth day, without a vesicle. The inaptitude for the disease, however, seldom prevails to this extent, and generally manifests itself in the difficulty of securing the infection; in the slow progress of the vesicle; or in some of those imperfections taken notice of hereafter.

From these different causes the disease may be so modified as to lose more or less of its protective influence. The inflammation may appear too early, and partake from the commencement, of the character of common inflammation; it may be interrupted in its progress; it may run its course too hastily and the scab fall off too soon; the vesicle may want the central depression, the pearl colour, or be filled with pus instead of lymph; the areola may be pale and indistinct, or may appear on the second day without a vesicle, large, irregular, itchy, and of a florid red color, disappearing before the sixth day.

It is frequently a subject of considerable solicitude to know whether this insusceptibility to the vaccine disease, affords evidence of a similar security against the contagion of small pox. To decide this question affirmatively, would require a degree of attention which has never been bestowed upon this point. Dr. Gregory maintains the affirmative, as the result of very extensive observation. But admitting the fact to be established so far as it applies to those cases of insusceptibility, which do not arise from any obvious disease liable to control the vaccine infection, a great number of cases will still remain, in which the insusceptibility to kine pox arises from diseases too feeble to control the small pox, or which, by co-operating with it, only add to its fatal character. When epidemics, arising from malaria, or atmospheric vicissitudes, prevail, they never fail to impart to the small pox their peculiar type and character. With regard to typhoid epidemics this fact has long been known, and we have seen, during the present summer, in an individual who had been exposed to the remote causes of intermittent fever on the Ohio River, the eruptive fever of smallpox assume distinctly the type of a bilious intermittent.

Not unconnected with this subject is the question, whether the security derived from vaccination diminishes with time. A very general impression of this kind prevails, and we regret to say there is reason to believe, has been encouraged by members of the medical profession. The universal belief, among those whose experience authorises them to express an opinion, is, that when the vaccine vesicle is perfect and uninterrupted in its progress, its protective influence continues through life. If the opinion be correct, that the susceptibility to the vaccine disease and the small pox are modified by the same circumstances, then individuals who, from some constitutional cause, have had the former in a modified and imperfect form, will as the causes which have produced this modification wear away, be liable to have the latter more or less modified, in proportion to the deviation of the vaccine vesicle from its genuine character.

These views are sustained by the fact that variola occurs at every period after vaccination, from a few days or weeks, to many years; and on the other hand, that numerous individuals have exposed themselves to the contagion of small pox with impunity, who derive their protection from vaccination, performed above twenty years ago.

What is the value of vaccination as a preventive of small pox? It can no longer be maintained by the most sanguine advocates for vaccination, that it is, at all times, an effectual security. The number of cases of small pox occurring after vaccination, first became so numerous as to attract the attention of the public in 1813. The cases so frequently met with were at first accounted for, by supposing the process of vaccination to have been imperfect ; and this explanation was, in some degree, confirmed, by the appearance of the scar and the history of the disease. The fact however, is now indubitably established by the concurrent evidence of physicians whose testimony cannot on any account be questioned, that small pox may follow vaccination, when the progress of the latter has been perfectly satisfactory.

When revaccination is resorted to, in most cases it will take no effect. When it does, the disease will sometimes run its usual course without modification ; but much more frequently it will assume some one of the various forms which we have enumerated above, among the varieties of imperfect vaccination. Somewhat analogous to this is the progress of small pox after vaccination. In a very few cases, the disease pursues its course without any modification. In other cases though the violence of the disease is undiminished, it runs its course more rapidly. In by far the greater number, the eruptive fever makes its attack with its customary violence, vesicles make their appearance, and disappear about the 5th or 6th day without suppurating ; so that the secondary fever, from which is the principal source of danger, never occurs.

The disease however, occasionally proves fatal in this modified form. 1st. In constitutions of great nervous irritability, or under circumstances calculated to produce great visceral congestion, the disease may prove fatal at its very onset. 2. The seeds of some fatal disease may be lurking in the system, ready to be called into fatal activity by any febrile disease. Under these circumstances it is true that small pox is but the exciting cause of the fatal disease, but from the difficulty of drawing the line of distinction between the effects of small pox and those of the superinduced disease, if the latter be at all rapid in its progress, the fatal event is generally attributed to the former disease. Yet even with these admissions, the number of fatal cases after satisfactory evidence of vaccination is very small. The greater number of cases reported as instances of this kind, are those in which the appearance of the scar, and the history of the vaccination are very unsatisfactory.

During the year 1827, 765 cases of small pox after variola, and after vaccine, are estimated to have occurred in Philadelphia.* Of these the larger number by far occurred in persons who had been previously vaccinated, leading at first view to the impression that the security derived from vaccination, is not so complete, as that from inoculation. But when we reflect that the number of vaccinated persons is far greater than of those who are protected by small pox; the inference drawn from this fact must be very much qualified. Admitting the fact that vaccination is less effectual as an entire preventative, it on the other hand has a much greater influence in controlling the violence of subsequent small pox. Thus of the cases reported to have occurred after vaccination, ten terminated fatally, but of these two only afforded satisfactory evidence of having passed through the genuine cow pox, and one of these was complicated with pneumonia typhodes—leaving but one death fairly referrible to small pox. Reports made from other sources place the subject in a rather more unfavorable light; but under any view of the case, the chances, in any one individual, against a fatal attack of small pox after genuine vaccination, are so great as to leave little room for individual solicitude. On the other hand, if the proportion of cases of secondary small pox be smaller, the disease is much more violent. Nine fatal cases are reported to have taken place in Philadelphia in 1827. Dr. Brincle, physician to the small pox hospital has at different periods met with 14 cases of secondary small pox, of which two died.

So far as these facts can be relied on, we may conclude that the security derived from small pox is very little greater than that

*See Report of the committee of Phil. Med. Society, appointed to collect facts in relation to the recent occurrence of the small pox.

from the vaccine disease ; and if we take into consideration the comparative violence of even inoculated small pox, the individual danger is increased, while the injury which may result to the community from introducing the infection of so fatal a disease, cannot be calculated. Both diseases, when genuine, may generally be considered as effectually securing the system against the small pox—both are liable, from causes of accidental or constitutional character, to lose their efficacy ; and probably there may be constitutions, in which the predisposition to small pox is so strong that one attack of the disease is insufficient to remove it. In confirmation of this last view, it has been repeatedly stated that secondary small pox most frequently occurs in those persons, whose persons bear the most unequivocal testimony to the violence of the former attack. Would not however, the analogy of the modification produced in the cow pox by violent inflammation, lead us to suppose that the same state of things may occur in variola, and that violent inflammation may so far change the specific character of the latter, as to impair or destroy its protecting influence ?

To what circumstance are we to attribute the increased number of failures, which are reported to have occurred since 1813.

With respect to the opinion, that the cow pox has lost its efficacy by passing through numerous individuals, we have already expressed our views. There is reason to believe that the varioloid has at different periods prevailed very extensively. The connection between the small pox and this disease was unknown until 1813, but an analogous disease, called nurse pox, is described by the older writers, as frequently occurring among the attendants upon those confined with the small pox ; and the chicken pox, with which the varioloid was once confounded, has been frequently mentioned as epidemic at the same time with small pox.

Much of the blame at present thrown upon vaccination, is to be attributed to the indifference of the public, and the carelessness and ignorance of those employed in vaccination. Dr. Jenner was employed many years in studying the subject before the results of his observations were made known to the world; and in the mean time, he had ascertained the true characteristics of the disease, with its various spurious forms, with as much precision as we know them at the present time. When the practice was established, it at once attracted a large share of public attention. The business of vaccinating was confided to the Medical profession, and the genuine character of the vesicle was made a subject of the closet investigation. The novelty of the remedy, and the recent experience of the small pox, rendered individuals anxious to avail themselves of its protecting influence, and induced government to make effectual provision for extending its benefits to every grade of society. To the contrast between this and the present state of feeling upon the subject, it is scarcely necessary to refer. Very many physicians scarcely think it necessary to make the characteristics of the vaccine disease a subject of particular investigation, and if they did, the scanty remuneration they receive (in this city at least,) does not compensate them for devoting any attention to the progress of the disease. Many are vaccinated by private persons, and the notions which prevail upon the subject are any thing but correct. We have repeatedly heard persons speak of a high degree of local inflammation, as a certain criterion of successful vaccination; at other times in children laboring under various cutaneous diseases, we have had unhealthy pustules pointed out to us, to shew that the vaccination "had taken finely." Under these circumstances it is not surprising that individuals should be disappointed, and the public confidence impaired in the remedy.

So far as vaccination is concerned, these facts tend to shew that vaccination is entitled to nearly all the credit it originally possessed. If it should appear that with these qualifications failures are still more frequent than formerly, and especially, that cases of secondary small pox are more numerous, something may perhaps be attributed to atmospheric causes. Small pox like other epidemics is disposed at one time to spread more rapidly, and prevail with more fatality than at others; in other words there is a wide spread constitution of the atmosphere favorable to the spread of small pox, and perhaps at these times the disease may attack individuals, who, under other circumstances would be protected from its aggression. We do not wish to be considered as perfectly satisfied with this explanation. We offer it merely as a probable key to difficulties, which at present are not well understood.

Of the different plans proposed with the view of providing against the uncertainty of vaccination, we shall mention but two, *inoculation* and *revaccination*.

In objecting to the first of these, we need only remark that it is a mode of protecting individuals, at the expense of the public safety. So nearly impossible is it to keep even an individual so secluded as to avoid communicating the infection, that the practice, under every precaution, must at all times, destroy more lives than it can possibly save. Since the modified small pox, therefore, is as infectious as the disease in its genuine form, it would imply a great perversion of moral feeling, to endanger the whole community for the purpose of guarding individuals against a remote contingency. Even when professional duty requires great personal exposure, a man of spirit would rather incur the consequences of the disease, than set the example for so pernicious a practice The only circumstances under which it would be justifiable are, when the vaccine infection cannot be communicated ; not for individual security, but for the purpose of testing the principle, whether the immunity also extends to the small pox.

Revaccination is a test satisfactory in its nature, and perfectly safe. But since full confidence, on the part of the public, in the security derived from vaccination, is essential to its universal application, the practice should be discountenanced except in doubtful cases. When these occur, vaccination should be repeated until the physician is satisfied that further precaution is unnecessary. It is necessary for the practitioner to be on his guard, lest the difficulty of communicating the infection, where a partial protection exists, should lead him to suspect an entire immunity.

Under the impression that the virus deteriorates from repeated vaccination, it has been proposed to renew the infection from the original source. Many difficulties lie in the way of the execution of this proposition. It is difficult to procure the infection from the animal; the cow is subject to other diseases so nearly similar, as to occasion much difficulty in drawing the distinction, and requiring repeated vaccinations to prove the genuineness of the disease; and lastly, the recent disease is more violent without affording greater security.

We presume that we have advanced sufficient to prove that vaccination, as it exists already, is sufficient to obviate all the dangers of the small pox, and if properly attended to, all the inconveniences of the modified disease, and even to extirpate them entirely. So far as the remedy depends upon the medical profession, their duty is obvious. Any remarks upon the policy which expediency may require of the civil authorities, does not comport with the course marked out for the Western Journal; but they may at some future time be made the subject of a communication to the public, through a different channel.

